# Use of a Western blot technique for the serodiagnosis of glanders

**DOI:** 10.1186/1746-6148-7-4

**Published:** 2011-01-19

**Authors:** Mandy C Elschner, Holger C Scholz, Falk Melzer, Muhammad Saqib, Peggy Marten, Astrid Rassbach, Michael Dietzsch, Gernot Schmoock, Vania L de Assis Santana, Marcilia MA de Souza, Renate Wernery, Ulrich Wernery, Heinrich Neubauer

**Affiliations:** 1 Friedrich Loeffler Institute, Federal Research Institute for Animal Health, Institute of Bacterial Infections and Zoonoses, Naumburger Strasse 96a, 07743 Jena, Germany; 2 Bundeswehr Institute of Microbiology, Neuherbergstrasse 11, 80937 Munich, Germany; 3 Veterinary Research Centre, PO Box 50 Seeb, Postal Code 121, Sultanate of Oman; 4 Department of Clinical Medicine and Surgery, University of Agriculture, Faisalabad 38040, Pakistan; 5 Laboratório Nacional Agropecuário, Ministério Da Agricultura, Rua Dom Manoel De Medeiros, S/N Dois Irmãos Recife PE Brasil CEP 52171-030, Brazil; 6 Central Veterinary Research Institute, Dubai PO Box 597, United Arabic Emirates

## Abstract

**Background:**

The in vivo diagnosis of glanders relies on the highly sensitive complement fixation test (CFT). Frequently observed false positive results are troublesome for veterinary authorities and cause financial losses to animal owners. Consequently, there is an urgent need to develop a test with high specificity. Hence, a Western blot assay making use of a partly purified lipopolysaccaride (LPS) containing antigen of three *Burkholderia mallei *strains was developed. The test was validated investigating a comprehensive set of positive and negative sera obtained from horses and mules from endemic and non endemic areas.

**Results:**

The developed Western blot assay showed a markedly higher diagnostic specificity when compared to the prescribed CFT and therefore can be used as a confirmatory test. However, the CFT remains the test of choice for routine testing of glanders due to its high sensitivity, its feasibility using standard laboratory equipment and its worldwide distribution in diagnostic laboratories.

**Conclusions:**

The CFT should be amended by the newly validated Western blot to increase the positive likelihood ratio of glanders serodiagnosis in non endemic areas or areas with low glanders prevalence. Its use for international trade of horses and mules should be implemented by the OIE.

## Background

Glanders, caused by *Burkholderia (B.) mallei*, is a highly contagious disease in equines which is notifiable to the World Organisation of Animal Health (OIE, Office International des Epizooties). The disease is still endemic in the Middle East, Asia and South America. Recent outbreaks have been reported from Turkey, the United Arabic Emirates, Iraq, Iran, India, Pakistan, Mongolia, China, Brazil and most recently from Bahrain [1-11]. The distribution of glanders in Africa is unknown.

The diagnosis of *B. mallei *infection still relies on serological proof by agglutination test and complement fixation test (CFT), or proof of the presence of a specific delayed hypersensitivity reaction after intracutaneous application of mallein [12,13]. The CFT for glanders is so far the only officially recognized serological test in international trade of equidae. The CFT has a sensitivity of at least 97% [14] but a notable number of unspecific, false positive results occur [15-18]. False positive results due to cross-reactions may be seen in horses suffering from strangles, equine influenza or petechial fever. The test can also not be applied on sera having so called "anticomplementary activity". In general, serological tests may be negative in emaciated and chronically debilitated animals suffering from glanders [16].

Glanders was eradicated from Western Europe, Australia and North America in the last century applying a rigorous culling of horses found positive in complement fixation and mallein test [15,19]. These techniques are useful in eradication programs with regard to specificity and sensitivity [12]. However, in glanders free areas or in areas with very low prevalence of glanders highly specific tests are needed to minimize the number of false positive results [20]. Lipopolysaccharide (LPS) preparations have already been applied in Western blot analysis or competition ELISA to detect anti-*B. mallei *antibodies in serum of horses [20-22]. But current protocols for the extraction and purification of LPS from *B. mallei *are time consuming, sophisticated and hazardous for the operator [23,24].

Here we describe a new Western blot assay based on an easy to prepare LPS preparation containing antigen from *B. mallei*, purified from soluble components. With a specificity and a sensitivity of 100%, the assay can be used to confirm positive and at the same time to exclude false positive CFT results.

## Methods

### Production of hyperimmune sera in rabbits

One rabbit was immunized with a crude suspension (10^6 ^cfu/ml) of heat inactivated *B. mallei, B. pseudomallei, B. cepacia, and Pseudomonas aeruginosa*, respectively. The strains used are given in Table 1. The first and second immunizations (days 0 and 3) were done by intracutaneous application of 10 doses of 0.2 ml (10^6 ^cfu/ml) antigen without adjuvant distributed on the back. Further, 5 immunizations were done by application of 0.5 ml (10^6 ^cfu/ml) in the ear vein (days 10, 17, 24, 31, and 38). Seroconversion was monitored by CFT and Western blot assay using the homologous antigen. The final sera were collected 6 weeks after the first immunization (day 42). The animal experiment was authorized by the government of Thuringia, Germany (registration number 04-106/07).

**Table 1:** List of bacterial strains used in this study

Strain	Source	Geographic origin	Species
Mukteswar	Horse	India	*Burkholderia mallei*
Bogor	Horse	Indonesia	*Burkholderia mallei*
Zagreb	Horse	Yugoslavia	*Burkholderia mallei*
ATCC 23343	Human	unknown	*Burkholderia pseudomallei*
DSM 7288	unknown	unknown	*Burkholderia cepacia*
ATCC 9027	unknown	unknown	*Pseudomonas aeruginosa*

ATCC: American Type Culture Collection, Manassas, USA

DSM: German Collection of Microorganisms and Cell Cultures, Braunschweig, Germany

### Immunisation of a horse

To produce a panel of positive control sera, one horse was immunized subcutaneously using a mixture of crude suspensions of heat inactivated *B. mallei *strains Mukteswar, Bogor and Zagreb (10^9 ^cfu/ml) adjuvanted with aluminium hydroxide gel (Sigma Chemie GmbH, Munich, Germany). Immunizations were performed weekly for seven weeks with 7 doses of 1.5 ml antigen containing increasing cell concentrations (10^3 ^cfu/ml to 10^9 ^cfu/ml). The titer was analysed in parallel by CFT and Western blot analysis twice a week and for 10 weeks after the first immunization. The animal experiment was authorized by the government of Thuringia, Germany (registration number 04-105/07).

### Samples

A total of 2,282 sera (group I) were collected from different horse populations in several geographical areas of Germany during 2006 to 2009.

These sera were considered negative for glanders because Germany is free of the disease for more than 50 years.

The specificity testing was carried out with 305 samples (group II) randomly selected from group I regarding the Mersenne Twister method by use of the Software SPSS (SPSS Inc., Chicago, USA).

The sensitivity of the Western blot assay was tested using 205 true positive sera (group III). Of these, 171 CFT positive samples were collected from microbiologically or clinically/mallein positive glanderous horses and mules in Punjab, Pakistan (n = 59), Pernambuco, Brazil (n = 87), and Dubai, United Arabic Emirates (n = 25). Included in group III were additionally 21 and 13 sera from the *B. mallei*-immunized horse and rabbit, respectively.

### CFT

The CFT was performed according to the instructions of the OIE Manual of Diagnostic tests and Vaccines for Terrestrial Animals [13] using a certified, commercially available antigen containing antigen from *B. mallei *strains Bogor, Mukteswar, and Zagreb (cc-pro GmbH, Oberdorla, Germany), and a complement and ready-to use hemolytic system (Institut Virion/Serion GmbH, Würzburg, Germany). Samples were considered negative when 100% haemolysis occurred at 1:5 dilution, suspicious when 25-75% haemolysis was seen at dilution 1:5 and positive when no haemolysis was detected at dilution 1:5.

### Antigen preparation for the Western blot assay

*B. mallei *strains Bogor, Zagreb and Mukteswar were grown on blood agar plates over night at 37°C under aerobic conditions. Details on the strains are given in Table 1. For LPS purification, a 10-microliter loop full of bacterial colony material was re-suspended thoroughly in 6 ml saline (0.9% NaCl, pH 7.0) up to a density comparable to McFarland scale 4.0. A volume of 3 ml of 37% formaldehyde was added to achieve a final formaldehyde concentration of 12.3%. The suspension was rigorously vortexed and subsequently swayed using an orbital shaker (Polymax 1040, Heidolph Instruments GmbH, Schwabach, Germany) over night at room temperature. Controls for sterility confirmed inactivation of bacteria. Cells were pelletized by centrifugation at 3.500 × g for 15 min and the supernatant was discarded. Soluble components were removed by 3 consecutive centrifugation and washing steps with 9 ml phosphate buffered saline (PBS, pH 7.0) shaking at 15 rpm for 10 minutes. The LPS-containing pellet was re-suspended in 9 ml PBS. Two further extraction steps by adding 1 volume of 37% formaldehyde into 2 volume re- suspended cells in PBS were applied. By 3 consecutive washing steps with PBS the soluble cell components and the formaldehyde were removed. After the last washing step the pellet was resuspended in PBS. Aliquots were directly used for SDS-polyacrylamide gel electrophoresis (SDS-PAGE) and subsequent Western blot analysis. The washing steps and the number of bacteria used for LPS preparation were crucial for the efficient removal of soluble components from the LPS-containing cell debris. Optimal purification was achieved using 9 × 10^8 ^cells/ml (McFarland scale 3) or less (data not shown).

**Figure 1:**
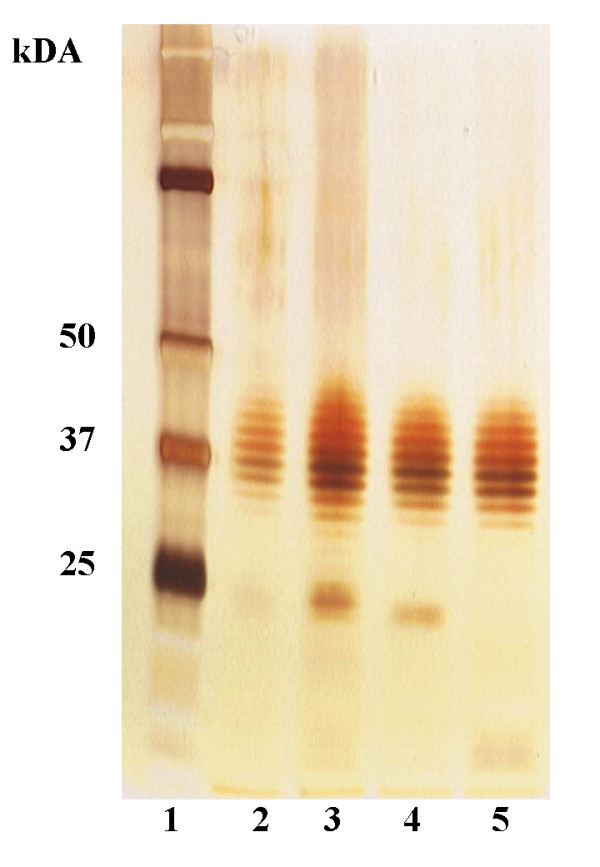
**Silver stained SDS-PAGE gel**. Silver stain of SDS-PAGE of mixtures of purified LPS of *B. mallei *strains protein standard (lane 1), Mukteswar (lane 2); Bogor (lane 3); Zagreb (lane 4), mixture of all (lane 5).

The correct formation of a typical LPS ladder was proofed by SDS-PAGE and silver staining before blotting. For SDS-PAGE several dilutions from the prepared LPS containing cell suspension in Laemmli Buffer (Sigma, Munich, Germany) were loaded on a precast denaturating 4-12% polyacrylamide gradient gel (Invitrogen, Karlsruhe, Germany) and separated at a constant voltage of 200V for 50 min using the Xcell SureLock™ MiniCell (Invitrogen). Silver staining was carried out with the SilverQuest™ silver staining kit (Invitrogen). The LPS extracted from each of the *B. mallei *strains showed the typical LPS ladder with no visible differences. We believe that soluble antigens are mainly removed from the preparation but we are well aware, that our antigen is not pure and contains other components, like high molecular weight components (Figure 1). The protein content of each antigen preparation was below 0.5 mg/ml, measured by absorption at 280 nm (Spectrophotometer NanoDrop 1000, Thermo Fisher Scientific, Bonn, Germany). For further use, the antigen preparations of the *B. mallei *strains Bogor, Zagreb and Mukteswar were mixed equally.

### Immuno blotting

**Figure 2:**
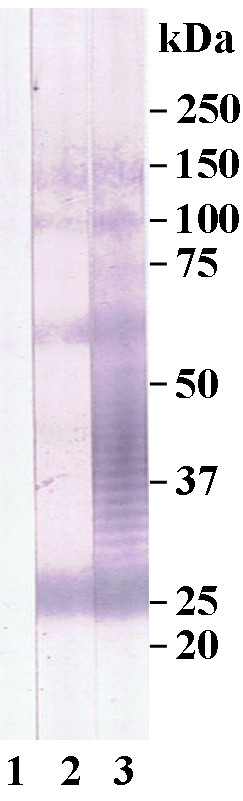
**Score of immunoblot**. Score of immunoblot: lane 1: negative, lane 2: suspicious, lane 3: positive.

For Western blotting analysis, 350 μl LPS-containing suspension of *B. mallei *strains Bogor, Zagreb and Mukteswar were separated on a precast preparative 4-12% polyacrylamide gradient gel (Invitrogen) as described above. LPS was transferred to a 0.45 μm nitrocellulose membrane (Invitrogen) by blotting at constant 30V for 1 h using the Novex^® ^Blot Module (Invitrogen). The membrane was blocked overnight in Blocking Solution (Candor Bioscience, Weißensberg, Germany). After three washing steps the membrane was cut into strips of 3 mm and stored at -20°C or was used for subsequent immunoblot analysis. Stripes were incubated with equine or rabbit sera in a 1:50 dilution in Low Cross Buffer (Candor Bioscience) for 1.5 h at room temperature followed by three washing steps in washing buffer (Candor Bioscience) 20 min each. The strips were then incubated for 1.5 h at room temperature in Low Cross Buffer containing alkaline phosphatase-conjugated rabbit anti-horse-IgG or goat anti-rabbit-IgG (Sigma, Munich, Germany, 1:5,000). After three additional washing steps, the strips were stained with NBT-BCIP^® ^solution (Sigma). The immunostaining was stopped by washing with distilled water after 10 minutes. The immunoblot was scored positive if the banding pattern of the *B. mallei *LPS ladder within the region of 20 to 60 kDa was clearly visible, scored suspicious if a weak color reaction was detected, and scored negative if no reaction was seen (Figure 2).

## Results

### CFT

All 205 samples of group III were positive resulting in a sensitivity of 100%. Seventy of the 1,282 sera of group I showed a false positive or suspicious CFT result resulting in a specificity of 94.5%.

### Western blot assay

**Figure 3:**
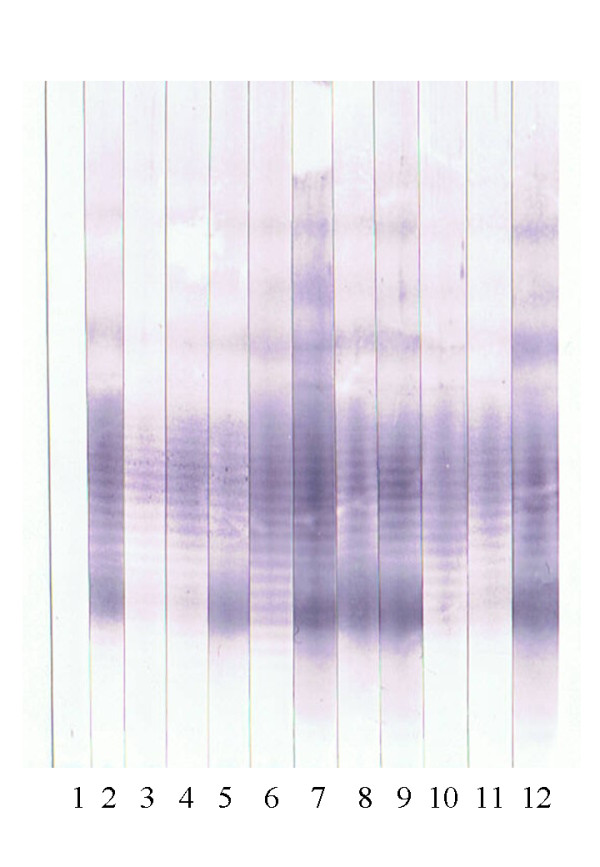
**Western blot analysis of sera from Pakistan**. Lane 1: negative control; lane 2: positive control; lane 3-12 sera of acutely infected horses from Pakistan.

All 305 samples of group II were tested negative and all of 205 positive samples (group III) showed a clearly visible LPS banding pattern and were scored positive. A panel of immunoblot strips of samples from Pakistan are shown in Figure 3. On this basis, the diagnostic sensitivity and specificity of the Western blot was calculated to be 100%. CFT false positive samples (n = 70) from group I were also tested negative.

**Figure 4:**
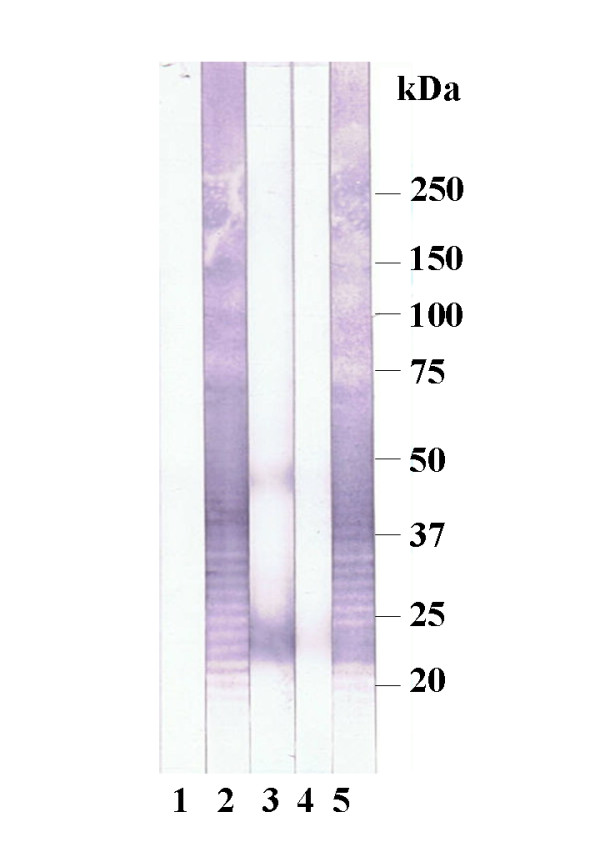
**Western blot analysis of rabbit hyperimmunesera (HIS) against *B. mallei *LPS**. Lane 1: negative control serum, lane 2: anti *B. mallei *HIS, lane 3: anti *B. cepacia *HIS, lane 4: anti *P. aeruginosa *HIS, lane 5: anti *B. pseudomallei *HIS.

Rabbit hyperimmune sera against *B. cepacia *and *P. aeruginosa *showed no clearly visible LPS banding pattern. Only the anti- *B. pseudomallei *serum cross reacted with the *B. mallei *antigen in the Western blot assay (Figure 4).

## Discussion

Following eradication of glanders from the Western hemisphere, false positive CFT results pose a considerable problem in trade of equids. The need for the development and evaluation of highly specific serological tests for exclusion of false positive results became imperative. One competitive ELISA (cELISA) was described based on mallein as antigen and an in house monoclonal antibody (mAb) [22]. Another cELISA used a commercially available *B. mallei *specific mAb (3D11) and a simple carbohydrate preparation of *B. mallei *as antigen [20]. Both tests show comparable sensitivity (98.9% and 98.6%, respectively) and a specificity of 100%. Due to the sophisticated technique, the high costs for mallein and mAbs and the restricted availability of these mAbs, the cELISAs will not be available for routine laboratories in the future. Other tests still used for diagnosing glanders are Rose Bengal agglutination test, indirect hemagglutination test, counter immunoelectrophoresis and indirect fluorescent antibody test [21,25-29] which have either technical shortcomings or have low specificity and/or sensitivity. New approaches using microarray technology for the serodiagnosis of glanders and melioidosis are based on polysaccharide antigens [30] but these techniques are expensive and not suited for routine mass testing of sera.

The CFT is used for diagnosis of many diseases worldwide and has a high sensitivity, especially when used for glanders serodiagnosis. This finding was again demonstrated by our study. We conclude therefore that the CFT is still the most valuable screening test currently available. But again, the CFT had a specificity of only 94.5% in a set of horse sera from a glanders free region (Germany). Consequently, a considerable number of CFT false positive tested animals must be expected when horses from glanders free regions are tested by the current OIE standards [6]. The need of the development of a supplementary confirmatory test with a high negative likelihood ratio for international trade was stressed again.

*B. mallei *LPS-based Western blot assays are cheap, easy to produce and user friendly test systems which have been developed in the past, but could not be evaluated on a significant number of positive and negative horse sera until now [12]. We were able to collect 205 sera of infected animals from geographically different regions and a set of sera from immunized animals (horse and rabbit), enabling us to validate the Western blot assay for its use in the routine serodiagnosis of glanders.

It was proven that the Western blot technique is highly specific and is able to supplement the CFT to avoid false positive diagnoses in horses and mules. The advance of the immunoblot technique is that specific bands can be distinguished from background reactions in contrast to the CFT. The investigation of the hyperimmune sera of rabbits against *B. mallei *LPS showed that *B. cepacia *and *P. aeruginosa *won't interfere with this technique. *B. pseudomallei *antibodies, however, will cause false positive results due to known cross-reacting epitopes of the LPS [31,32]. In international trade, it is also of utmost importance not to transport animals infected with the dangerous zoonotic agent *B. pseudomallei *from endemic areas to areas with ambient environment where *B. pseudomallei *could establish a temporal or permanent reservoir. An identification and destruction of those animals will have an additional zoosanitary and ecological benefit.

## Conclusions

The developed method of LPS-preparation reported here is highly feasible and cheap. The qualitative and quantitative composition of our antigen proofed to be suitable for its use in immunobloting. The developed method is a highly sensitive and specific serological test for glanders diagnosis. It will be of great value in glanders endemic areas in less developed countries.

## Abbreviations

CFT: complement fixation test; LPS: lipopolysaccaride; OIE: Office International des Epizooties (World organisation for animal health); Cfu: colony forming units; PBS: phosphate buffered saline; SDS-PAGE: sodium dodecylsulfate polyacrylamide gel electrophoresis; mAb: monoclonal antibody; cELISA: competitive enzyme linked immunosorbent assay; ATCC: American Type Culture Collection; DSM: German Collection of Microorganisms and Cell Cultures

## Authors' contributions

MCE designed the study, coordinated the investigation of all samples by CFT and western blot assay, evaluated and interpreted the data and wrote the manuscript.

HN has the supervision of the research group, contributed to acquisition of funding, co-design of the study and was involved in the drafting of the manuscript.

HCS, HN established the antigen purification procedure and westernblot assay.

UW, RW, MS, VLAS, MMAS have been involved in malleinisation and health monitoring of the horses, collection and pre-testing and providing of samples including the interpretation of results.

PM established, standardized and performed the western blot assay.

FM, AR, MD, GS have been involved in the design of the study, providing and supporting laboratory work and discussion of results including drafting the manuscript.

All authors revised the manuscript critically and approved the final version.
